# Autocrine activin A signalling in ovarian cancer cells regulates secretion of interleukin 6, autophagy, and cachexia

**DOI:** 10.1002/jcsm.12489

**Published:** 2019-08-21

**Authors:** Kristine Pettersen, Sonja Andersen, Anna van der Veen, Unni Nonstad, Shinji Hatakeyama, Christian Lambert, Estelle Lach‐Trifilieff, Siver Moestue, Jana Kim, Bjørn Henning Grønberg, Alain Schilb, Carsten Jacobi, Geir Bjørkøy

**Affiliations:** ^1^ Department of Biomedical Laboratory Science, Faculty of Natural Sciences NTNU‐Norwegian University of Science and Technology Trondheim Norway; ^2^ Centre of Molecular Inflammation Research, Department of Clinical and Molecular Medicine NTNU‐Norwegian University of Science and Technology Trondheim Norway; ^3^ Department of Circulation and Medical Imaging, Faculty of Medicine NTNU‐Norwegian University of Science and Technology Trondheim Norway; ^4^ Department of Cancer Research and Molecular Medicine NTNU‐Norwegian University of Science and Technology Trondheim Norway; ^5^ Clinic of Oncology St. Olavs Hospital ‐ Trondheim University Hospital Trondheim Norway; ^6^ Novartis Institutes for BioMedical Research Basel, Musculoskeletal Disease Area Novartis Pharma AG Basel Switzerland

**Keywords:** Cachexia, Autophagy, IL‐6, Activin, Autocrine loop

## Abstract

**Background:**

The majority of patients with advanced cancer develop cachexia, a weight loss syndrome that severely reduces quality of life and limits survival. Our understanding of the underlying mechanisms that cause the condition is limited, and there are currently no treatment options that can completely reverse cachexia. Several tumour‐derived factors and inflammatory mediators have been suggested to contribute to weight loss in cachectic patients. However, inconsistencies between studies are recurrent. Activin A and interleukin 6 (IL‐6) are among the best studied factors that seem to be important, and several studies support their individual role in cachexia development.

**Methods:**

We investigated the interplay between activin A and IL‐6 in the cachexia‐inducing TOV21G cell line, both in culture and in tumours in mice. We previously found that the human TOV21G cells secrete IL‐6 that induces autophagy in reporter cells and cachexia in mice. Using this established cachexia cell model, we targeted autocrine activin A by genetic, chemical, and biological approaches. The secretion of IL‐6 from the cancer cells was determined in both culture and tumour‐bearing mice by a species‐specific ELISA. Autophagy reporter cells were used to monitor the culture medium for autophagy‐inducing activities, and muscle mass changes were evaluated in tumour‐bearing mice.

**Results:**

We show that activin A acts in an autocrine manner to promote the synthesis and secretion of IL‐6 from cancer cells. By inhibiting activin A signalling, the production of IL‐6 from the cancer cells is reduced by 40–50% (up to 42% reduction on protein level, *P* = 0.0048, and 48% reduction on mRNA level, *P* = 0.0308). Significantly reduced IL‐6 secretion (*P* < 0.05) from the cancer cells is consistently observed when using biological, chemical, and genetic approaches to interfere with the autocrine activin A loop. Inhibiting activin signalling also reduces the ability of the cancer cells to accelerate autophagy in non‐cancerous cells (up to 43% reduced autophagy flux, *P* = 0.0006). Coherent to the *in vitro* data, the use of an anti‐activin receptor 2 antibody in cachectic tumour‐bearing mice reduces serum levels of cancer cell‐derived IL‐6 by 62% (from 417 to 159 pg/mL, *P* = 0.03), and, importantly, it reverses cachexia and counteracts loss of all measured muscle groups (*P* < 0.0005).

**Conclusions:**

Our data support a functional link between activin A and IL‐6 signalling pathways and indicate that interference with activin A‐induced IL‐6 secretion from the tumour has therapeutic potential for cancer‐induced cachexia.

## Introduction

Cancer cachexia (hereafter referred to as cachexia) severely compromises quality of life and limits survival of up to 80% of patients with advanced cancer.^1^ Cachexia is characterized by a substantial loss of lean body mass that is often, but not always, accompanied by loss of fat.^2^ Currently, no therapeutic approach consistently reverses cachexia in humans. The development of therapeutic strategies has been slowed by the incomplete understanding of the underlying mechanisms that lead to the condition.

Several inflammatory and tumour‐derived factors are suggested to drive development of cachexia. One of these is activin A, a member of the transforming growth factor beta (TGFβ) superfamily of proteins. Activin A administration alone is sufficient to cause weight loss (including muscle loss) in mice.^3^ Also, α‐inhibin‐deficient mice (lacking a central inhibitor of activin) lose weight.^4^ Furthermore, inhibition of activin receptor 2B (ActRIIB), a receptor for a subset of TGFβ members including activin A, reverses cachexia and increases survival in several mouse models.^5^ Elevated activin A levels in sera from pancreatic cancer patients are associated with shorter survival.^6^ This might relate to development of cachexia.

It has been suggested that several members of the TGFβ superfamily, including activin A, can affect skeletal muscle by limiting the differentiation of myoblasts.^7^, ^8^ Moreover, the transcription factors SMAD2 and SMAD3, which are activated by, for instance, activin A, can induce atrophy in adult myofibers, in a mechanism involving the AKT/mammalian target of rapamycin pathway.^9^ Although convincing data underscores a role of activin A in cachexia development, other signalling substances and pathways are likely also involved.

Several studies point to tumour‐derived interleukin 6 (IL‐6) as a central driver of cachexia. We have shown that IL‐6 is secreted from cancer cells, and, via trans‐signalling, it can accelerate the catabolic process autophagy in muscle cells, thereby contributing to cachexia.^10^ Also, IL‐6 administration is sufficient to drive cachexia,^11^ and IL‐6‐blocking agents can reverse muscle loss and increase survival in mice.^12^, ^13^, ^14^ Recently, it was shown that the human ovarian cancer cell line ES‐2 induces cachexia in mice in an IL‐6‐dependent manner.^15^ Moreover, increased IL‐6 levels have been detected in serum from cachectic patients,^16^ and importantly, case studies in humans show promising results when blocking the IL‐6 receptor, using the neutralizing antibody tocilizumab.^17^, ^18^

The existence of convincing data with regard to the cachexia‐causing ability of both activin A and IL‐6 could be due to different mechanisms operating in different contexts and patients but could also be due to a functional relationship between activin A and IL‐6. In order to investigate this, we have used the ovarian carcinoma cell line TOV21G. These cells secrete high amounts of activin A and cause a rapid development of cachexia at a low tumour burden in mice.^5^, ^10^ In this model, cachexia can be reversed by injecting activin decoy receptor,^5^ thereby supporting a role for activin A in TOV21G‐driven cachexia. We recently reported that the TOV21G cells, in addition to activin A, secrete high levels of IL‐6.^10^ This makes TOV21G a good tool for testing whether there is a functional relationship between activin A and IL‐6 in cachexia development. Here, we present data demonstrating that autocrine activin A signalling in cancer cells is needed for their release and systemic distribution of IL‐6. We also show that activin A‐induced IL‐6 secretion from these cells stimulates autophagy in non‐cancerous cells, a catabolic process suggested by us^10^ and others^19^, ^20^, ^21^, ^22^ to be involved in the pathogenesis of cachexia. Furthermore, we find that interference with activin A signalling in cachectic tumour‐bearing mice reduces serum levels of cancer cell‐derived IL‐6 and reverses cachexia. Collectively, our results point to a novel mechanism by which both activin A and IL‐6 can contribute to cachexia and that this interplay may provide targets for treatment of cancer cachexia.

## Methods

### Reagents

During *in vitro* cell culture experiments, the following reagents were used as specified: Recombinant (r) human/mouse/rat activin A (R&D Systems, 338‐AC‐010/CF), rHuman IL‐6 (Invitrogen, cat. no. PHC0066), rHuman Activin RIIB Fc Chimera (R&D Systems 339RB/CF), human/mouse/rat activin A (beta A subunit) antibody (R&D Systems, MAB3381), ALK4/5/7 inhibitor (Selleck Chem, cat. no. SB431542), 3‐methyladenine (Sigma, M9281), bafilomycin A1 (Sigma, B1793), Torin 1 (Cayman Chemicals, #10997‐10), and Hanks' Balanced Salt Solution (Sigma, H9269).

CDD866 is a chimeric murinized version of BYM338/bimagrumab, an anti‐ActRII antibody, where the human Fc region of the antibody has been replaced by a mouse Fc (IgG2a Leu234Ala/Leu235Ala), provided by the Novartis Biologic Units.

### Cell culture

Cells were maintained in a humidified atmosphere of 5% CO_2_ and 95% air at 37°C. TOV21G cells from ATCC (CRL‐11730) were cultured in 42.5% medium 199 + GlutamaxTMI (Gibco 41150) and 42.5% medium MCDB 105 [Sigma M6395, dissolved in dH_2_O to 1 L final volume, added NaHCO3 (1.5 g/L), and adjusted to pH 7.3], supplemented with foetal bovine serum (FBS, 15%) and gentamicin (0.05 mg/mL). HEK293 green fluorescent protein (GFP)‐p62 cells (a kind gift from Prof. T. Johansen and colleagues) were cultured in DMEM (Sigma D5796) supplemented with FBS (10%) and gentamicin (0.05 mg/mL).

### RNA interference

For inhibin beta A (INHBA), activin receptor 2A (ACVR2A), activin receptor 2B (ACVR2B), KRAS, and SMAD3 downregulation, TOV21G cells were transfected using 20 nM siRNA (final concentrations), and DharmaFECT transfection reagent 1 (Dharmacon) was diluted in Opti‐MEM 1 Reduced Serum Medium (#31985‐070, Gibco Life Technologies). After 24 h, the transfection medium was exchanged for normal growth medium, and the cells were incubated for 1 or 3 days before conditioned medium (CM) was harvested and protein and RNA were isolated. At this time, cultures were approximately 80% confluent. The following siRNA oligonucleotides were obtained from Dharmacon: L‐011701‐00‐0010 ON‐TARGETplus SMARTpool human INHBA, L‐004926‐00‐0010 ON‐TARGETplus SMARTpool human ACVR2A, L‐004927‐00‐0010 ON‐TARGETplus SMARTpool human ACVR2B, L‐020067‐00‐0005 ON‐TARGETplus SMARTpool human SMAD3, and D‐001210‐01‐20 non‐targeting siRNA. Two siRNA oligonucleotides targeting KRAS were obtained from Ambion: cat. no. 4390824 (ID s7939 and ID s7940).

### Immunoblotting

#### SMAD3 signalling in autophagy reporter cells

HEK293 autophagy reporter cells were exposed to 50 μg/mL recombinant activin A for 5 min at the end of 2 h incubation in normal growth medium containing 0.1% FBS. Cell were lysed by cell scraping on ice in a buffer containing 8 M urea, 0.5% (v/v) Triton X‐100, 100 mM DTT, 1xComplete® protease inhibitor, and 8% phosphatase inhibitor cocktail I and III (Sigma). Protein concentration was determined by BioRad protein assay (BioRad). Equal amounts of proteins were separated using NuPAGE® Novex® 4‐12% Bis‐Tris Gels (Invitrogen) and dry blotted on nitrocellulose membranes. The membrane was blocked, and antibodies were diluted in a 1:1 mixture of Odyssey blocking buffer (Li‐Cor) and TBST (20 mM Tris, pH 7.6, 137 mM NaCl with 0.1 % Tween 20). Bound antibodies were imaged by near infrared fluorescence using appropriate fluorescent dye labelled secondary antibodies and an Odyssey NIR scanner (Li‐Cor Biosciences). Images were processed using the Li‐Cor Odyssey software image studio 2.0. The antibodies used for immunostaining were pSMAD3 (Ser423/Ser425) (Abcam, ab52903), diluted 1:1000, and beta‐tubulin (Abcam, ab6046), diluted 1:5000.

#### NF‐κB and p38 MAP kinase signalling in TOV21G after interfering with activin signalling

TOV21G cells were transfected with siRNA as described earlier. Three days after removal of transfection reagents, the cells were harvested in urea buffer, and extracts were applied on gels, blotted, and used for western detection as described earlier. Antibodies used for immunostaining were pNF‐κB p65 (Ser536) (Cell Signaling, #3033), phospho‐p38 MAP kinase (Thr180/Tyr182) (Cell Signaling, #9215), and Erk1/2 (Cell Signaling #9107), all diluted 1:1000.

### Autophagy reporter system

Autophagy was quantified using flow cytometry measuring GFP fluorescence in live HEK293 cells expressing GFP‐sequestosome 1 (SQSTM1) fusion gene, as previously described.^23^ HEK293 autophagy reporter cells were seeded in 24‐well plates (25 000 cells per well) and incubated for 1 day. Thereafter, the cells were treated as indicated for 3 days (unless otherwise specified) in the presence of doxycycline (1 ng/mL) before assessment of fusion protein degradation by flow cytometry. When using known autophagy effectors, these were added 17 h before assessment of fusion protein degradation. Protein degradation was determined as the loss of green fluorescent signal compared with control. Triplicate wells were used in all experiments, and signal was measured in 10 000 cells per well.

### ELISA

The levels of IL‐6 and activin A in CM from TOV21G cells were determined using human IL‐6 OptEIA ELISA kit (BD Biosciences, cat. no. 555220) and a human/mouse/rat activin A Quantikine ELISA kit (R&D Systems, cat. no. DAC00B), respectively. All analyses were performed according to the manufacturer's protocols.

The levels of human IL‐6 and murine IL‐6 in serum from mice were determined using the U‐PLEX Human IL‐6 Assay (Meso Scale Discovery, cat. no. K151TXK) and the U‐PLEX Mouse IL‐6 Assay (Meso Scale Discovery, cat. no. K152TXK) according to the protocols provided by the manufacturer. The levels of activin A in serum from mice were determined using a self‐made activin A ELISA, which was established according to the protocol of Meso Scale Discovery. The following antibodies were used for this ELISA: anti‐activin A primary antibody (R&D Systems, cat. no. MAB3381; detects human/rat/mouse activin A); biotinylated anti‐activin A secondary antibody (R&D Systems, cat. no. BAM3381; detects human/rat/mouse activin A); for detection, Sulfo‐TAG streptavidin (Meso Scale Discovery, cat. no. R32AD, 0.25 μg/mL); and for the titration of the standard curve, recombinant human activin A (R&D Systems, cat. no. 338 AC/CF). The antibodies in this sandwich ELISA are directed against the same epitope of the INHBA subunit and thus will detect only the homodimer (activin A). They do not recognize the inhibin beta B subunit, and accordingly, there is no cross reactivity towards activin AB. Due to low homology (18%) between INHBA and the alpha subunit found in inhibin A, any cross reactivity towards inhibin A is considered unlikely.

All presented ELISA data are within the range of the respective standard curves (*Figure* S1). When necessary, the samples were diluted to fall within the range of the standard curve.

### RNA isolation and quantitative real‐time PCR

TOV21G cells transfected with NT, INHBA, ACVR2A, ACVR2B, KRAS, or SMAD3 siRNA were left for 1 or 3 days post‐transfection (as explained earlier) before RNA was isolated using RNeasy Mini Kit (Qiagen, cat. no. 74106) according to the protocol from the manufacturer. Purity, quality, and concentration of isolated RNA were confirmed using Nanodrop. cDNA for qPCR analysis was made by using the QuantiTect Reverse Transcription Kit (Qiagen, cat. no. 205311) according to the manufacturer's protocol. mRNA levels were normalized against GAPDH. qPCR was performed in parallel 20 μL reactions containing 10 μL Perfecta qPCR Fast‐Mix ROX (from Quanta, QUNT95077‐012), 1 μL of the respective primer set, and 9 μL template (5 and 25 ng RNA for GAPDH and the remaining primer sets, respectively). The following TaqMan primer sets from Life Technologies were used: human INHBA (Hs01081598_m1, cat. no. 4331182), human AVCR2A (Hs00155658_m1, cat. no. 4331182), human ACVR2B (Hs00609603_m1, cat. no. 4331182), human IL‐6 (Hs00985639_m1, cat. no. 4331182), human KRAS (Hs00364284_g1, cat. no. 4331182), human SMAD3 (Hs00969210_m1, cat. no. 4331182), and human GAPDH (Hs99999905_m1, cat. no. 4331182). The cycling conditions for the StepOne plus system (Applied Biosystems, Foster City, CA, USA) were 45°C for 2 min, 95°C for 30 s, and 40 cycles of 95°C for 1 s and 65°C for 20 s. Relative RNA transcription levels were transformed into linear form by 2^−ΔΔCt^.

### Animal experiments

The animal experiment was performed according to the regulations effective in the Canton of Basel City, Switzerland, under the license number BS‐2186. Eight‐week‐old female mice (Hsd:Athymic Nude‐*Foxn1* ^*nu*^) were purchased from Harlan Laboratories (Horst, Netherlands), acclimated for 7 days housed at 25°C with a 12:12 h light–dark cycle, and provided *ad libitum* water and a standard laboratory diet containing 18.2% protein and 3.0% fat with an energy content of 15.8 MJ/kg (NAFAG 3890, Kliba, Basel, Switzerland).

TOV21G cells were harvested by trypsin treatment and suspended in a solution containing 50% PBS and 50% BD Matrigel™ matrix without phenol red (cat. no. 356237, BD Biosciences, Bedford, MA) at a density of 3 × 10^7^/mL. A 0.1 mL of cell suspension containing 3 × 10^6^ cells was inoculated subcutaneously into the left flank of mice anaesthetized with Forane® (isoflurane; Abbott AG, Baar, Switzerland) using a Station anaesthesia minihub (Tem Sega, Pessac, France). When tumours were palpable, length and width of tumours were measured through skin, and tumour volumes were calculated according to the formula (length × width^2^)/2. Seven days after cell inoculation, mice bearing tumours with acceptable morphology and size (mean volume of approximately 130 mm^3^) were randomized to groups containing 10 mice per group, and the treatments were initiated on the day of randomization (Day 0). CDD866 was administered at 20 mg/kg s.c., once weekly in a volume of 5 mL/kg. Body weight and tumour volume were measured two to three times per week. At the end of the experiment (Day 35), the mice were euthanized with CO_2_. In the group of vehicle‐treated mice, three mice had to be sacrificed at Days 28, 30, and 32, respectively, for animal welfare reasons. For all animals, tumour, tibialis anterior, gastrocnemius–soleus–plantaris complex, quadriceps, heart, liver, kidney, perigonadal white adipose tissue, spleen, and brain were collected and weighed. The brain weight was measured for normalization of organ weights.^24^

Before performing our analyses, we omitted three mice from each of the two groups inoculated with TOV21G cells due to very large or small tumours (larger or smaller than average ±SD).

### cBioPortal

cBioPortal (http://www.cbioportal.org/) is an open‐access database that allows visualization and analysis of large‐scale cancer genomics data sets.^25^, ^26^ Our analyses utilize the co‐expression visualization to identify co‐expression of IL‐6 and INHBA mRNA. For the analysis, we used the TCGA Provisional data set for ovarian serous cystadenocarcinoma.

### Statistics

Statistical work was performed using SPSS or Microsoft Excel. The statistical tests performed are specified when individual results are presented.

### Data presentation

All figures were mounted using Canvas 14 (ACD Systems).

## Results

### Activin A promotes secretion of autophagy‐accelerating factors from tumour cells

We have previously found that weight loss in cancer patients is associated with autophagy‐inducing bioactivity in the blood stream.^10^ These findings implicate a causal role in cachexia development for tumour‐derived signalling substances that increase autophagy in normal cells. We have shown that IL‐6 is abundantly secreted from certain cancer cells and potently accelerates autophagy in muscle cells when complexed to soluble IL‐6 receptor.^10^ This way of IL‐6 signalling may contribute to weight loss. Because tumour‐derived activin A is proposed to be a mediator of cancer cachexia,^3^, ^5^ we hypothesized that also activin A contributes to cachexia by accelerating autophagy in host cells.

**Figure 1:**
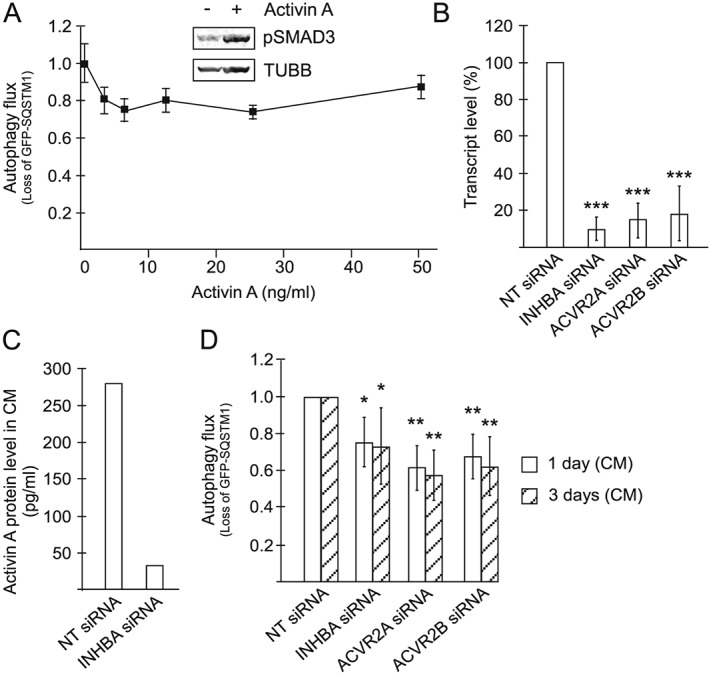
Activin A acts in an autocrine or paracrine loop to promote the secretion of autophagy‐accelerating compounds from TOV21G cells. (A) Lower panel: Autophagy flux in autophagy reporter cells treated with recombinant activin A for 3 days at indicated concentrations. Mean from three independent experiments, each using triplicate wells ±SD. Upper panel: Level of phosphorylated SMAD3 (Ser423/Ser425) in autophagy reporter cells following 5 min exposure to activin A (50 ng/mL). β‐Tubulin is used as a loading control. (B) INHBA, ACVR2A, and ACVR2B transcript levels in TOV21G cells treated with siRNA targeting INHBA, ACVR2A, and ACVR2B, respectively, 1 day post‐transfection, relative to non‐targeting (NT) siRNA‐treated TOV21G cells. Measured using qPCR. Mean from six independent experiments ±SD. ^***^ *P* < 0.0005 vs. NT siRNA (Student's *t*‐test). (C) Level of activin A protein in CM from TOV21G cells treated with INHBA siRNA, 3 days post‐transfection. Mean from two experiments. (D) Autophagy flux in autophagy reporter cells treated with CM from TOV21G cells. CM had been collected 1 or 3 days post‐transfection using NT siRNA or siRNA targeting INHBA, ACVR2A, or ACVR2B. Mean from six independent experiments, each using triplicate wells. ^*^ *P* < 0.05, ^**^ *P* < 0.005 vs. respective NT siRNA (Student's *t*‐test).

To quantify autophagic flux, we utilized an autophagy reporter cell system,^23^ HEK293 cells with an inducible expression of GFP fused to SQSTM1 (also known as p62). SQSTM1 is specifically targeted by autophagy, and its time‐dependent decline (and consequently GFP decline/loss of green fluorescence) is a measure of autophagic activity and can be detected by flow cytometry. The autophagy reporter cells responded to recombinant activin A, evident as increased SMAD3 phosphorylation (*Figure* 1A, upper panel). However, activin A did not accelerate autophagy at any of the tested concentrations (*Figure* 1A, lower panel). This could mean that activin A contributes to cachexia in a manner that is independent of autophagy in host cells. Alternatively, activin A can still accelerate autophagy *in vivo* but in an indirect manner by influencing the abundancy of other autophagy‐inducing factors.

In mice, cachexia is reversed by activin receptor antagonists.^5^, ^27^ This may be due to the ability of these agents to prevent activin A‐induced signalling in muscle cells (and other host cells). Alternatively, the receptor antagonist could inhibit activin A signalling in the tumour and affect the secretion of other cachexia‐inducing factor(s). Because we observed no direct effect of activin A on autophagy, we hypothesized that activin A may control the secretion of other factors that accelerate autophagy and cause cachexia.

TOV21G ovarian cancer cells have been shown to secrete high amounts of activin A.^5^ Moreover, we have shown that CM from these cells potently accelerates autophagy in other cells,^10^ and, as shown by us^10^ and others,^5^ TOV21G tumours potently cause weight loss in mice. Importantly, cachexia in these mice can be reversed by interfering with activin A signalling.^5^ To test if autocrine signalling could affect the autophagy‐inducing activity of the CM, activin A signalling was targeted by siRNA directed towards either INHBA or ACVR2A/ACVR2B (also called ACTRIIA/ACTRIIB, respectively). INHBA encodes the INHBA subunit that is needed to form bioactive activin A homodimers. Activin A initiates signalling via type 1 and 2 activin receptors and of the type 2 receptors activin A can bind to both ActRIIA and B.^28^ All siRNAs efficiently reduced the level of their target transcripts (*Figure* 1B), and the reduction in mRNA levels was maintained for at least 3 days post‐transfection (data not shown). No apparent effects of the different siRNAs were noted on cell proliferation or survival (data not shown). Consistent with the depletion of the target transcripts, INHBA siRNA reduced the activin A protein secreted by TOV21G by about 90% (from an average of 278 to 32 pg/mL), measured by ELISA (*Figure* 1C). Having successfully depleted components of autocrine activin A signalling, we asked whether this reduced the autophagy‐accelerating ability of CM from the cells. When using the autophagy reporter cell system, we found that the CM from TOV21G cells treated with INHBA, ACVR2A, or ACVR2B siRNA was clearly less potent in accelerating autophagy as compared with CM from cells treated with non‐targeting siRNA. Approximately 40% reduced autophagy flux was observed already 1 day post‐transfection, and this effect was sustained for at least 3 days (*Figure* 1D). By comparison, the autophagy inhibitors 3‐methyladenine or bafilomycin A1 (17 h) reduce the autophagy flux in the reporter cells by 30% and 40%, respectively (*Figure* S2). Our results indicate that although activin A may not activate autophagy directly, it can act in an autocrine or paracrine manner to promote secretion of other autophagy‐accelerating factors from the cancer cells.

### Activin A is important for secretion of the autophagy‐accelerating factor IL‐6 from cancer cells

**Figure 2:**
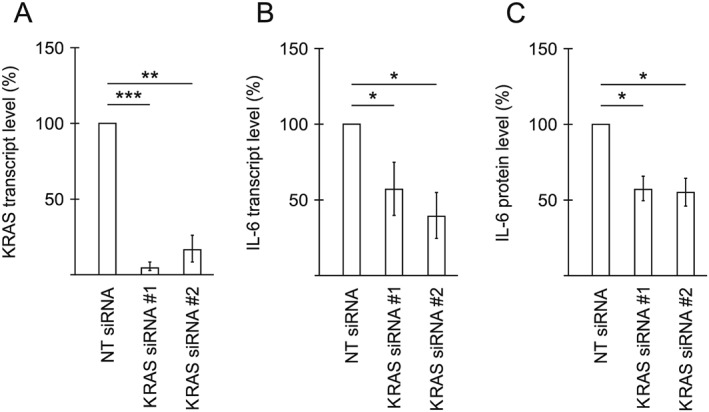
KRAS affects the secretion of the autophagy‐inducing cytokine IL‐6 from TOV21G cells. (A) KRAS transcript level in TOV21G cells treated with two different siRNAs targeting KRAS, 1 day post‐transfection, relative to non‐targeting (NT) siRNA‐treated TOV21G cells. Measured using qPCR. Mean from three independent experiments ±SD. ^***^ *P* < 0.0005 and ^**^ *P* < 0.005 vs. NT siRNA (Student's *t*‐test). (B) IL‐6 transcript level in TOV21G cells treated with siRNAs targeting KRAS, 1 day post‐transfection, relative to NT siRNA‐treated TOV21G cells. Measured using qPCR. Mean from three independent experiments ±SD. ^*^ *P* < 0.05 vs. NT siRNA (Student's *t*‐test). (C) Relative level of IL‐6, measured using an ELISA assay, in CM from TOV21G cells treated with NT siRNA or siRNAs targeting KRAS. CM was harvested 1 day post‐transfection. Mean from three experiments ±SD. ^*^ *P* < 0.05 vs. NT siRNA (Student's *t*‐test).

**Figure 3:**
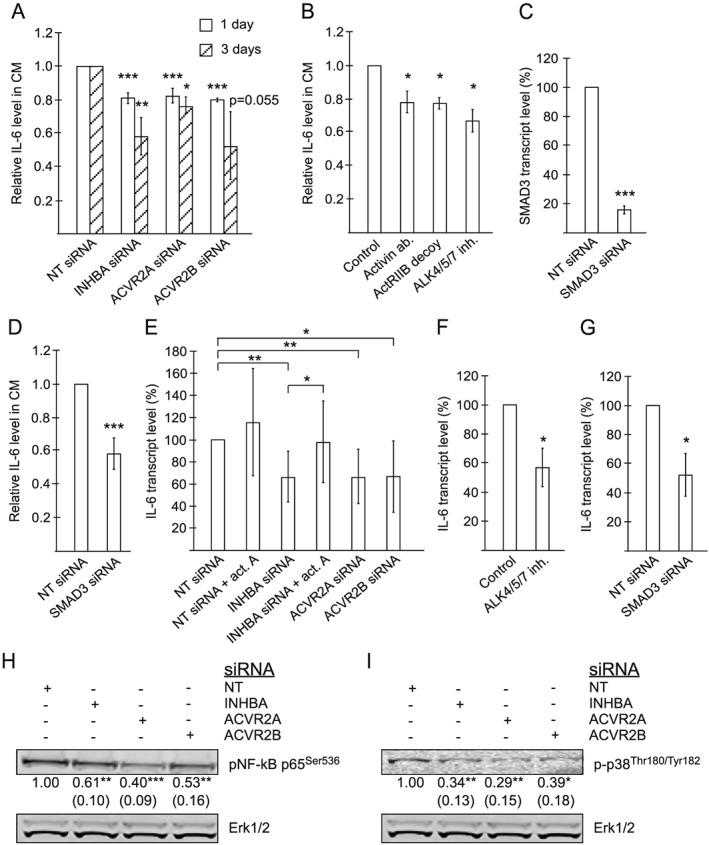
Activin A affects the secretion of the autophagy‐inducing cytokine IL‐6 from TOV21G cells. (A) Relative level of IL‐6, measured using an ELISA assay, in CM from TOV21G cells treated with non‐targeting (NT) siRNA or siRNA targeting INHBA, ACVR2A, or ACVR2B. CM was harvested 1 or 3 days post‐transfection. Mean from three experiments [ACVR2A (3 days) and ACVR2B (1 day and 3 days)], four experiments [ACVR2A (1 day) and INHBA (3 days)], or six experiments [INHBA (1 day)] ±SD. ^*^ *P* < 0.05, ^**^ *P* < 0.005, ^***^ *P* < 0.0005 vs. respective NT siRNA (Student's *t*‐test). (B) Relative level of IL‐6, measured using an ELISA assay, in CM from TOV21G cells treated with 1.6 μg/mL activin neutralizing antibody, 1.8 μg/mL ActRIIB decoy receptor, or 10 μM ALK4/5/7 inhibitor (SB431542). CM was harvested after 1 day of treatment. Mean from three experiments ±SD. ^*^ *P* < 0.05 vs. control (Student's *t*‐test). (C) SMAD3 transcript level in TOV21G cells treated with siRNA targeting SMAD3, 1 day post‐transfection, relative to NT siRNA‐treated TOV21G cells. Measured using qPCR. Mean from three independent experiments ±SD. ^***^ *P* < 0.0005 vs. NT siRNA (Student's *t*‐test). (D) Relative level of IL‐6, measured using an ELISA assay, in CM from TOV21G cells treated with NT siRNA or siRNA targeting SMAD3. CM was harvested 1 day post‐transfection. Mean from six experiments ±SD. ^***^ *P* < 0.0005 vs. NT siRNA (Student's *t*‐test). (E) IL‐6 transcript level in TOV21G cells treated with NT siRNA (±50 ng/mL recombinant activin A) or siRNA targeting INHBA (±50 ng/mL recombinant activin A), ACVR2A, or ACVR2B. Measured using qPCR. RNA was isolated 1 or 3 days post‐transfection. Mean from 10 independent experiments (seven for recombinant activin A) ±SD. ^*^ *P* < 0.05, ^**^ *P* < 0.005 (Student's *t*‐test). (F) IL‐6 transcript level in TOV21G cells treated with 10 μM ALK4/5/7 inhibitor (SB431542) for 1 day, relative to vehicle‐treated TOV21G cells. Measured using qPCR. Mean from three independent experiments ±SD. ^*^ *P* < 0.05 vs. vehicle (Student's *t*‐test). (G) IL‐6 transcript level in TOV21G cells treated with siRNA targeting SMAD3, 1 day post‐transfection, relative to NT siRNA‐treated TOV21G cells. Measured using qPCR. Mean from three independent experiments ±SD. ^*^ *P* < 0.05 vs. NT siRNA (Student's *t*‐test). (H, I) Levels of pNF‐κB p65 (Ser536) and Erk1/2, and p‐p38 (Thr180/Tyr182) and Erk1/2, respectively, in TOV21G cells treated with NT siRNA or siRNA targeting INHBA, ACVR2A, or ACVR2B. Extracts were made 3 days post‐transfection. Representative blots are shown with numbers representing mean values from five [panel (H)] or four [panel (I)] independent experiments (and SD) after normalization against signals from Erk1/2. ^*^ *P* < 0.05, ^**^ *P* < 0.005, ^***^ *P* < 0.0005 (Student's *t*‐test).

We have previously found that the CM from TOV21G cells accelerates autophagy in other cells and have largely attributed this effect to their secretion of IL‐6.^10^ TOV21G cells are known to have an activating KRAS mutation, and this has been shown to be important for IL‐6 production, especially in murine lung cancer models.^29^, ^30^ We therefore wanted to establish whether activated KRAS was a major contributor to the IL‐6 production in our model system. Two different siRNAs for KRAS were shown to very efficiently take down the level of KRAS transcripts (*Figure* 2A), and this was accompanied by clearly reduced levels of IL‐6 transcripts in the cells (*Figure* 2B). Also, IL‐6 protein in CM 1 day post‐transfection was reduced about 43% from an average of 23.5 ng/mL in CM from non‐targeting siRNA transfected cells (*Figures* 2C and S3). To investigate if activin‐mediated signalling could also influence IL‐6 production, we performed siRNA‐mediated knockdown of either INHBA or ACVR2A/ACVR2B in TOV21G cells. Indeed, all siRNAs caused reduced levels of IL‐6 in the CM (about 20% reduction from an average of 23.5 ng/mL in CM from non‐targeting siRNA transfected cells), as measured by an IL‐6 ELISA 1 day post‐transfection (*Figures* 3A and S3) and confirmed by a bioplex assay (data not shown). The effect was sustained for at least 3 days post‐transfection (24–47% reduction, depending on the siRNA, from an average of 49.1 ng/mL in CM from non‐targeting siRNA transfected cells) (*Figures* 3A and S3).

Consistent with an autocrine function of activin A in the regulation of IL‐6 secretion from the cancer cells, an activin A neutralizing antibody reduced the level of IL‐6 in CMs by 22% from 6.1 ng/mL in untreated cells (*Figures* 3B and S3). It should be noted that this antibody is directed towards the INHBA subunit that is also found in activin AB,^31^ potentially also recognizing activin AB. However, based on transcriptome characterization (data not shown), the expression level of the beta B subunit in these cells is very low (below detection limit), indicating that activin AB is not of any significance in this system. Further, an ActRIIB decoy receptor and an ALK4/5/7 inhibitor reduced IL‐6 levels in CM by 23% and 33%, respectively, from 6.1 ng/mL in untreated cells (*Figures* 3B and S3). Moreover, SMAD3 siRNA efficiently reduced the level of SMAD3 transcripts and significantly reduced the secretion of IL‐6 from TOV21G cells (42% reduction from an average of 23.5 ng/mL in CM from non‐targeting siRNA transfected cells) (*Figures* 3C and 3D and S3), showing that canonical activin A signalling, involving ALK4/5/7‐induced SMAD3, is important for the induced secretion of IL‐6.

Because autocrine activin A signalling clearly stimulates IL‐6 secretion from the cancer cells, we asked if this involved increased transcription of the IL‐6 gene. qPCR analysis of NT, INHBA, ACVR2A, and ACVR2B siRNA‐treated TOV21G cells demonstrated that all siRNAs targeting activin A signalling caused reduced mRNA level of IL‐6, supporting that autocrine activin A signalling promotes IL‐6 transcription (*Figure* 3E). In accordance, ALK4/5/7 inhibition or siRNA‐mediated SMAD3 knockdown reduced the level of IL‐6 transcripts (*Figure* 3F and 3G). Importantly, treatment with recombinant activin A could significantly counteract the effect of INHBA siRNA on IL‐6 transcript level (*Figure* 3E), demonstrating that the effects on IL‐6 transcription are regulated by autocrine activin A signalling. The IL‐6 gene is among the typical NF‐κB controlled genes. Thus, we asked if the autocrine activin A‐induced IL‐6 transcription could be controlled by NF‐κB. Interference with activin signalling using siRNAs causes a clear reduction in the levels of pNF‐κB (p65), which is in accordance with the effects observed on IL‐6 transcript levels (*Figure* 3H). NF‐κB has been shown to collaborate with p38 MAPK to induce IL‐6 expression.^32^ Also, p38 MAPK is one of the signalling mediators downstream of activin A.^33^ We found that coinciding with reduced pNF‐κB and IL‐6 levels, interference with activin A signalling reduced the level of phospho‐p38 in the cancer cells (*Figure* 3I). These data suggest that p38 MAPK and NF‐κB play a role in activin A‐induced production of IL‐6.

### Interference with activin A signalling reduces tumour‐derived IL‐6 in sera and reverses cachexia in mice

Our results clearly demonstrate that autocrine activin A signalling in the cancer cell culture promotes the release of IL‐6. Thus, we tested whether activin A antagonism is able to reduce the systemic (serum) level of IL‐6 in tumour‐bearing animals and whether a putative reduction in IL‐6 level is associated with reversal of cachexia. We treated TOV21G tumour‐bearing mice with an ActRII neutralizing antibody (CDD866). CDD866 efficiently inhibits the binding of ligands, including activin A and myostatin, to both ActRIIA and ActRIIB.^27^, ^34^

**Figure 4:**
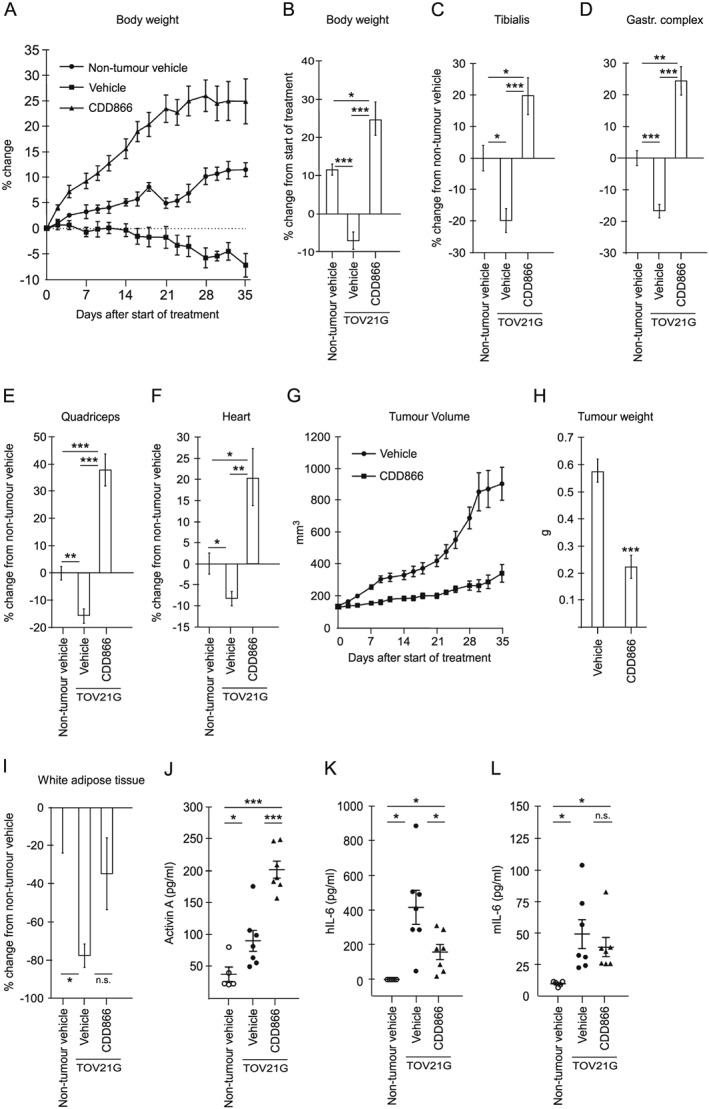
Interference with activin A signalling reduces tumour‐derived IL‐6 in sera and reverses cachexia in mice. (A, B) Mean relative body weight (at indicated time intervals) and mean relative change in body weight ±SEM, respectively, of non‐tumour control mice (*n* = 5) and vehicle‐treated (*n* = 7) and CDD866‐treated (*n* = 7) TOV21G tumour‐bearing mice. For the vehicle group, the data on Days 30, 32, and 35 are based on six, five, and four mice, respectively. (C–F) Mean relative change in tibialis, gastrocnemius complex, quadriceps, and heart weight, respectively, ±SEM of vehicle‐treated (*n* = 7) and CDD866‐treated (*n* = 7) TOV21G tumour‐bearing mice relative to non‐tumour control mice (*n* = 5). (G, H) Mean tumour volume and tumour weight ±SEM, respectively, of vehicle‐treated (*n* = 7) and CDD866‐treated (*n* = 7) TOV21G tumour‐bearing mice. (I) Mean relative change in white adipose tissue weight ±SEM of vehicle‐treated (*n* = 7) and CDD866‐treated (*n* = 7) TOV21G tumour‐bearing mice relative to non‐tumour control mice (*n* = 5). (J–L) Activin A, human IL‐6 (hIL‐6), and murine IL‐6 (mIL‐6) protein level, respectively, in sera from non‐tumour control mice (*n* = 5) and vehicle‐treated (*n* = 7) and CDD866‐treated (*n* = 7) TOV21G tumour‐bearing mice. Mean ± SEM are indicated. ^*^ *P* < 0.05, ^**^ *P* < 0.005, ^***^ *P* < 0.0005 (Student's *t*‐test), n.s. = non‐significant.

As expected, the TOV21G tumour‐bearing mice rapidly developed cachexia, evident as loss of body weight, muscle (tibialis, gastrocnemius complex, quadriceps, and heart), and white adipose tissue (*Figure* 4A–4I). Importantly, CDD866 treatment clearly reversed cachexia; the mice gained body weight, heart, and muscle mass, even to an extent that surpassed weight and muscle mass of non‐tumour‐bearing mice (*Figure* 4A–4F). Similar observations were also made for kidney and liver, while there was no significant change in the weight of spleen after the CDD866 treatment (*Figure* S4). Of note, CDD866 treatment also led to reduced tumour size (*Figure* 4G and 4H). Similar to the observed reversal of loss of muscle, the CDD866‐treated mice also seemed to have reduced loss of white adipose tissue; however, this effect was not significant (*P* = 0.06) (*Figure* 4I).

As anticipated, TOV21G‐bearing mice had increased serum level of activin A, and an additional rise in serum activin A was detected following CDD866 treatment (*Figure* 4J). By the use of species‐specific ELISAs, we also found that tumour‐bearing mice were subjected to a massive rise in the level of serum IL‐6 derived from the cancer cells (human IL‐6) (*Figure* 4K) and a significant, but clearly less substantial, increase in host‐derived murine IL‐6 (*Figure* 4L). Because activin A was important for IL‐6 secretion from the cancer cells *in vitro*, we tested whether the ActRII neutralizing antibody (CDD866) could reduce the level of IL‐6 in sera from TOV21G‐bearing mice. In accordance with our *in vitro* findings, inhibition of ActRII caused a significant reduction of cancer‐derived human IL‐6 in serum from the tumour‐bearing mice (*Figure* 4K). Interestingly, despite the increase in mouse IL‐6 levels in serum, treatment with the ActRII neutralizing antibody did not significantly reduce this pool of IL‐6 (*Figure* 4L). Together, this shows that disrupting activin A signalling in the tumour decreases systemic abundance of tumour‐derived IL‐6 and coincides with reversal of cachexia in the mice.

### Association between INHBA and IL‐6 gene expression in human ovarian tumours

**Figure 5:**
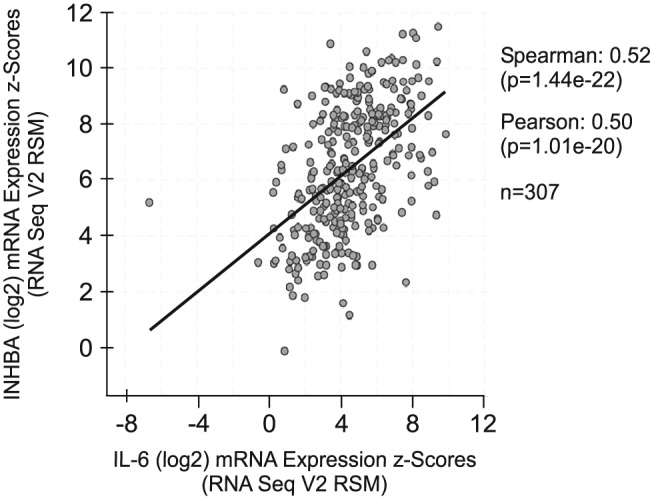
Association between INHBA and IL‐6 gene expression in human ovarian tumours. IL‐6 and INHBA mRNA expression in human ovarian serous cystadenocarcinoma (*n* = 307). From the cBioPortal database (http://www.cbioportal.org/).

Finding that activin signalling is needed for release of IL‐6 *in vitro* and in the TOV21G murine cachexia model, we investigated the possible association between activin A and IL‐6 in human ovarian cancer patients. By evaluating the levels of transcripts encoding IL‐6 and INHBA in tumour biopsies from ovarian cancer patients (*n* = 307) using the cBioPortal website, we found a significant association between the expression of these genes (*Figure* 5). This indicates that activin A may also modulate the expression of IL‐6 in human tumours.

## Discussion

Activin A and IL‐6 are both suggested to contribute to cachexia, and accordingly, their individual role in development of this condition has been a central topic of research. In mouse models of cancer‐induced cachexia, it has been demonstrated that both activin A and IL‐6 are contributing, and neutralizing each axis reverses the muscle wasting.^5^, ^11^, ^12^, ^13^, ^14^, ^27^, ^35^, ^36^, ^37^, ^38^, ^39^ Here, we show for the first time that intratumoural activin A signalling promotes IL‐6 secretion from the cancer cells, thus demonstrating a direct link between the two factors. We show that interference with activin A signalling reduces IL‐6 transcription, secretion, and systemic distribution. Reduced IL‐6 levels following obstruction of activin A activities associate with reduced ability of the cancer cells to accelerate autophagy in non‐cancerous cells *in vitro* and the ability to cause cachexia in mice. This study presents a so far unexplored mechanism to drive cachexia that may be targeted in treatment.

The importance of intratumoural signalling activities in cachexia development has been highlighted also by others. Johnston *et al*.^40^ demonstrated that TWEAK/Fn14 signalling in the tumour, rather than the host, is important to cause cachexia in mice and that interference with these factors increases survival. Similar to our findings, the authors suggest that Fn14 stimulates the secretion of cachexia‐inducing factors from the tumour. However, the secreted cachexia‐inducing factor(s) were not identified. In hepatic stellate cells, the Fn14 ligand TWEAK has been shown to upregulate both transcription and secretion of inflammatory cytokines, including IL‐6.^41^ This suggests that TWEAK and activin A may both work in a paracrine or autocrine manner in the tumours to stimulate IL‐6 secretion from cancer cells and facilitate the development of cachexia.

TWEAK‐induced IL‐6 involves activation of NF‐κB.^42^ Similarly, we find that interfering with the autocrine activin signalling causes a clear reduction in the active form of NF‐κB. Furthermore, SMAD3 seems to be involved in this pathway, and preliminary data from our lab indicate that SMAD3 is upstream of activation of NF‐κB. Such a sequential order of events is in line with data from mouse keratinocytes where TGFβ‐induced Alk5 and SMAD3 are required for NF‐κB‐dependent gene expression.^43^ Interestingly, Qin *et al*. showed binding of SMAD4 to the IL‐6 promoter after TGFβ stimulation of ovarian surface epithelial cells.^44^ This suggests that SMAD signalling may also directly control IL‐6 gene expression.

The involvement of activin A in IL‐6 secretion has also been described in non‐cancerous cells, like mouse neutrophils, where the cells in response to LPS secrete IL‐6 in an activin A/SMAD3‐dependent manner.^45^ IL‐6 may act systemically and has been suggested to be one of the major contributors to cachexia.^12^, ^13^, ^14^, ^17^, ^18^, ^46^, ^47^, ^48^ We have shown that IL‐6 accelerates autophagy^10^ and this may in part explain the potent cachexia‐inducing effect of this cytokine. Consistently, we could demonstrate that interference with autocrine activin A signalling in the cancer cells is sufficient to reduce the ability of the cells to accelerate autophagy in non‐cancerous cells.

Our *in vitro* data show that activin A acts in an autocrine or paracrine manner to promote secretion of IL‐6 from the cancer cells. Due to the heterogeneous nature of a tumour *in vivo*, with a variety of infiltrating cell types, it is likely that activin A signalling also occurs between cancer cells and stromal cells. Such crosstalk could potentially contribute to a higher IL‐6 secretion than what can be caused by the transformed cancer cells themselves. Activin A may also act systemically to induce secretion of IL‐6 from other cell types located distantly from the tumour. For instance, muscle cells may secrete IL‐6,^49^ and an effect of activin A on IL‐6 levels may occur as a result of myocyte signalling. In line with this, preliminary data from our lab show that activin A can induce release of IL‐6 from cardiomyocytes *in vitro* (data not shown). Surprisingly, we found that although host IL‐6 levels increased in sera of cachectic tumour‐bearing mice, the levels were not significantly affected by interference with activin A signalling. It should, however, be noted that we used immune‐compromised nude mice with subcutaneous flank implantation of TOV21G, similarly to Zhou *et al*. who reported the ability of TOV21G to cause cachexia in an activin A‐driven mechanism.^5^ We acknowledge, however, that activin A may affect both cancer cell‐derived and host‐derived IL‐6 differently in the presence of a complete immune system and when the cancer cells are growing in the pelvic/abdominal cavity.

In this study, we used TOV21G cells to induce cancer cachexia. In a recent paper, Pin *et al*. used another ovarian clear cell carcinoma cell line, ES‐2, and argued that this is more similar to human ovarian cancer.^15^ The ES‐2 cell line also gives cachexia, and bioactive IL‐6 seems to be causal. A mechanism for IL‐6 regulation in the ES‐2 model has not yet been proposed, but activation of the RAS/RAF/MEK/ERK signalling pathway is a common feature of the two cell models. While TOV21G cells harbour activated KRAS, ES‐2 have activated BRAF.^15^ Consistently, we find that IL‐6 secretion from the TOV21G cells is driven by activin A and oncogenic RAS. Whether activin A and/or BRAF is involved in the IL‐6 secretion from ES‐2 is not known.

In our mouse model, we find that treating tumour‐bearing mice with an ACVR2 neutralizing antibody not only reverts muscle mass to normal level but also causes a significant increase in muscle mass. Possibly, this relates to the ability of this antibody to block the binding of both activin A and myostatin to the ACVR2.^27^, ^34^, ^50^ Myostatin deficiency has previously been shown to be highly anabolic.^51^, ^52^ Whether myostatin also has a role in regulating IL‐6 secretion, and thereby is influencing cachexia development, cannot be excluded but is beyond the scope of this study.

A complicating factor when interpreting the reduced hIL‐6 levels in circulation after interfering with the ACVR2 is that the tumour size is significantly influenced. The reduced levels of tumour‐derived IL‐6 in circulation could thus, to an unknown extent, be a result of reduced number of IL‐6‐producing TOV21G cells. Regardless, *in vitro*, we show that there is a direct effect of activin A signalling on IL‐6 gene transcription and IL‐6 protein secretion from the cancer cells. It is likely that this mechanism also is of relevance in the *in vivo* model.

Based on results from murine models, recent clinical trials have aimed to unravel the efficiency of blocking activin signalling in patients with cachexia or muscle dystrophy. Clinical trials involving ACVR2B decoy receptors, however, were terminated due to severe adverse effects, including bleeding.^53^ More specific antibodies directed towards the ACVR2B are being tested in clinical trials^53^, ^54^, ^55^; however, because ACVR2 are central receptors for multiple TGFβ family members, and our study points to the central role of activin A, specific inhibition of activin A itself may prove less invasive and equally efficient.

The present study provides novel insights into the interplay between activin A and IL‐6 secretion from cancer cells that may be important for the development of future therapeutic strategies to reverse cancer cachexia.

## Conflict of interest

None declared.

## Funding

This study was financially supported by grants from The Norwegian Cancer Society, The Research Council of Norway through its Centres of Excellence funding scheme (223255/F50), and The Research Council of Norway (139610/300).

## Supporting information


**Figure S1**. Standard curves from activin A and IL‐6 ELISA. Standard curve for activin A (A) and IL‐6 (B) ELISA used when analyzing TOV21G conditioned medium, and for activin A ELISA used for analyzing serum samples (C).

**Figure S2**. Autophagy flux in autophagy reporter cells treated with autophagy effectors. Autophagy flux in autophagy reporter cells treated for 17 hours with 3‐methyladenine (3‐MA, 3 mM), bafilomycin A1 (BafA1, 100 nM), Hanks' Balanced Salt Solution (HBSS) or Torin 1 (150 nM).

**Figure S3**. IL‐6 protein level in conditioned medium (CM) from TOV21G cells. Level of IL‐6 in CM from untreated TOV21G cells and TOV21G cells treated with non‐targeting (NT) siRNA. CM from 1 or 3 days post‐seeding (untreated) or post‐transfection (NT siRNA treated).

**Figure S4**. In TOV21G tumor‐bearing mice, injection of the ActRII neutralizing antibody does not affect the weight of the spleen but display an anabolic effect on kidney and liver. Mean relative weight change of spleen, kidney and liver, respectively ±SEM of vehicle‐treated (n = 7) and CDD866‐treated (n = 7) TOV21G tumor‐bearing mice relative to non‐tumor, vehicle‐treated control mice (n = 5). *p < 0.05, ***p < 0.0005 (Student t‐test), n.s. = non‐significant.

## References

[1] von Haehling S , Anker SD . Prevalence, incidence and clinical impact of cachexia: facts and numbers—update 2014. J Cachexia Sarcopenia Muscle 2014;5:261–263. 25384990 10.1007/s13539-014-0164-8 PMC4248411

[2] Fearon K , Strasser F , Anker SD , Bosaeus I , Bruera E , Fainsinger RL , et al. Definition and classification of cancer cachexia: an international consensus. Lancet Oncol 2011;12:489–495. 21296615 10.1016/S1470-2045(10)70218-7

[3] Chen JL , Walton KL , Winbanks CE , Murphy KT , Thomson RE , Makanji Y , et al. Elevated expression of activins promotes muscle wasting and cachexia. FASEB J: official publication of the Federation of American Societies for Experimental Biology 2014;28:1711–1723. 10.1096/fj.13-245894 24378873

[4] Matzuk MM , Finegold MJ , Mather JP , Krummen L , Lu H , Bradley A . Development of cancer cachexia‐like syndrome and adrenal tumors in inhibin‐deficient mice. Proc Natl Acad Sci U S A 1994;91:8817–8821. 8090730 10.1073/pnas.91.19.8817 PMC44697

[5] Zhou X , Wang JL , Lu J , Song Y , Kwak KS , Jiao Q , et al. Reversal of cancer cachexia and muscle wasting by ActRIIB antagonism leads to prolonged survival. Cell 2010;142:531–543. 20723755 10.1016/j.cell.2010.07.011

[6] Togashi Y , Kogita A , Sakamoto H , Hayashi H , Terashima M , de Velasco MA , et al. Activin signal promotes cancer progression and is involved in cachexia in a subset of pancreatic cancer. Cancer Lett 2015;356( Pt B:819–827. 25449777 10.1016/j.canlet.2014.10.037

[7] Trendelenburg AU , Meyer A , Jacobi C , Feige JN , Glass DJ . TAK‐1/p38/nNFκB signaling inhibits myoblast differentiation by increasing levels of Activin A. Skelet muscle 2012;2:3. 22313861 10.1186/2044-5040-2-3 PMC3295657

[8] Trendelenburg AU , Meyer A , Rohner D , Boyle J , Hatakeyama S , Glass DJ . Myostatin reduces Akt/TORC1/p70S6K signaling, inhibiting myoblast differentiation and myotube size. Am J Physiol Cell Physiol 2009;296:C1258–C1270. 19357233 10.1152/ajpcell.00105.2009

[9] Sartori R , Milan G , Patron M , Mammucari C , Blaauw B , Abraham R , et al. Smad2 and 3 transcription factors control muscle mass in adulthood. Am J Physiol Cell Physiol 2009;296:C1248–C1257. 19357234 10.1152/ajpcell.00104.2009

[10] Pettersen K , Andersen S , Degen S , Tadini V , Grosjean J , Hatakeyama S , et al. Cancer cachexia associates with a systemic autophagy‐inducing activity mimicked by cancer cell‐derived IL‐6 trans‐signaling. Sci Rep 2017;7:2046. 28515477 10.1038/s41598-017-02088-2 PMC5435723

[11] Bonetto A , Aydogdu T , Jin X , Zhang Z , Zhan R , Puzis L , et al. JAK/STAT3 pathway inhibition blocks skeletal muscle wasting downstream of IL‐6 and in experimental cancer cachexia. Am J Physiol Endocrinol Metab 2012;303:E410–E421. 22669242 10.1152/ajpendo.00039.2012 PMC3423125

[12] Strassmann G , Fong M , Freter CE , Windsor S , D'Alessandro F , Nordan RP . Suramin interferes with interleukin‐6 receptor binding in vitro and inhibits colon‐26‐mediated experimental cancer cachexia in vivo. J Clin Invest 1993;92:2152–2159. 8227330 10.1172/JCI116816 PMC288393

[13] Strassmann G , Fong M , Kenney JS , Jacob CO . Evidence for the involvement of interleukin 6 in experimental cancer cachexia. J Clin Invest 1992;89:1681–1684. 1569207 10.1172/JCI115767 PMC443047

[14] Tamura S , Ouchi KF , Mori K , Endo M , Matsumoto T , Eda H , et al. Involvement of human interleukin 6 in experimental cachexia induced by a human uterine cervical carcinoma xenograft. Clin Cancer Res 1995;1:1353–1358. 9815931

[15] Pin F , Barreto R , Kitase Y , Mitra S , Erne CE , Novinger LJ , et al. Growth of ovarian cancer xenografts causes loss of muscle and bone mass: a new model for the study of cancer cachexia. J Cachexia Sarcopenia Muscle 2018;9:685–700. 30009406 10.1002/jcsm.12311 PMC6104117

[16] Martignoni ME , Kunze P , Hildebrandt W , Kunzli B , Berberat P , Giese T , et al. Role of mononuclear cells and inflammatory cytokines in pancreatic cancer‐related cachexia. Clin Cancer Res 2005;11:5802–5808. 16115919 10.1158/1078-0432.CCR-05-0185

[17] Hirata H , Tetsumoto S , Kijima T , Kida H , Kumagai T , Takahashi R , et al. Favorable responses to tocilizumab in two patients with cancer‐related cachexia. J Pain Symptom Manage 2013;46:e9–e13. 23602326 10.1016/j.jpainsymman.2013.01.009

[18] Ando K , Takahashi F , Motojima S , Nakashima K , Kaneko N , Hoshi K , et al. Possible role for tocilizumab, an anti‐interleukin‐6 receptor antibody, in treating cancer cachexia. J Clin Oncol Off J Am Soc Clin Oncol 2013;31:e69–e72. 10.1200/JCO.2012.44.2020 23129740

[19] Aversa Z , Pin F , Lucia S , Penna F , Verzaro R , Fazi M , et al. Autophagy is induced in the skeletal muscle of cachectic cancer patients. Sci Rep 2016;6:30340. 27459917 10.1038/srep30340 PMC4962093

[20] Penna F , Baccino FM , Costelli P . Coming back: autophagy in cachexia. Curr Opin Clin Nutr Metab Care 2014;17:241–246. 24535215 10.1097/MCO.0000000000000048

[21] Penna F , Costamagna D , Pin F , Camperi A , Fanzani A , Chiarpotto EM , et al. Autophagic degradation contributes to muscle wasting in cancer cachexia. Am J Pathol 2013;182:1367–1378. 23395093 10.1016/j.ajpath.2012.12.023

[22] Tardif N , Klaude M , Lundell L , Thorell A , Rooyackers O . Autophagic‐lysosomal pathway is the main proteolytic system modified in the skeletal muscle of esophageal cancer patients. Am J Clin Nutr 2013;98:1485–1492. 24108784 10.3945/ajcn.113.063859

[23] Larsen KB , Lamark T , Overvatn A , Harneshaug I , Johansen T , Bjorkoy G . A reporter cell system to monitor autophagy based on p62/SQSTM1. Autophagy 2010;6:784–793. 20574168 10.4161/auto.6.6.12510

[24] Sellers RS , Morton D , Michael B , Roome N , Johnson JK , Yano BL , et al. Society of Toxicologic Pathology position paper: organ weight recommendations for toxicology studies. Toxicol Pathol 2007;35:751–755. 17849358 10.1080/01926230701595300

[25] Cerami E , Gao J , Dogrusoz U , Gross BE , Sumer SO , Aksoy BA , et al. The cBio cancer genomics portal: an open platform for exploring multidimensional cancer genomics data. Cancer Discov 2012;2:401–404. 22588877 10.1158/2159-8290.CD-12-0095 PMC3956037

[26] Gao J , Aksoy BA , Dogrusoz U , Dresdner G , Gross B , Sumer SO , et al. Integrative analysis of complex cancer genomics and clinical profiles using the cBioPortal. Sci Signal 2013;6:pl1. 23550210 10.1126/scisignal.2004088 PMC4160307

[27] Hatakeyama S , Summermatter S , Jourdain M , Melly S , Minetti GC , Lach‐Trifilieff E . ActRII blockade protects mice from cancer cachexia and prolongs survival in the presence of anti‐cancer treatments. Skeletal muscle 2016;6:26. 27462398 10.1186/s13395-016-0098-2 PMC4960708

[28] Wakefield LM , Hill CS . Beyond TGFβ: roles of other TGFβ superfamily members in cancer. Nat Rev Cancer 2013;13:328–341. 23612460 10.1038/nrc3500 PMC7608560

[29] Miller A , McLeod L , Alhayyani S , Szczepny A , Watkins DN , Chen W , et al. Blockade of the IL‐6 trans‐signalling/STAT3 axis suppresses cachexia in Kras‐induced lung adenocarcinoma. Oncogene 2016;36:3059. 27893707 10.1038/onc.2016.437

[30] Caetano MS , Zhang H , Cumpian AM , Gong L , Unver N , Ostrin EJ , et al. IL6 blockade reprograms the lung tumor microenvironment to limit the development and progression of K‐ras‐mutant lung cancer. Cancer Res 2016;76:3189–3199. 27197187 10.1158/0008-5472.CAN-15-2840 PMC4891282

[31] Morikawa M , Derynck R , Miyazono K . TGF‐β and the TGF‐β Family: Context‐Dependent Roles in Cell and Tissue Physiology. Cold Spring Harb Perspect Biol 2016;8. 10.1101/cshperspect.a021873 PMC4852809 27141051

[32] Craig R , Larkin A , Mingo AM , Thuerauf DJ , Andrews C , McDonough PM , et al. p38 MAPK and NF‐κB Collaborate to Induce Interleukin‐6 Gene Expression and Release. J Biol Chem 2000;275:23814–23824. 10781614 10.1074/jbc.M909695199

[33] Ding H , Zhang G , Sin KW , Liu Z , Lin RK , Li M , et al. Activin A induces skeletal muscle catabolism via p38β mitogen‐activated protein kinase. J Cachexia Sarcopenia Muscle 2017;8:202–212. 27897407 10.1002/jcsm.12145 PMC5377410

[34] Morvan F , Rondeau JM , Zou C , Minetti G , Scheufler C , Scharenberg M , et al. Blockade of activin type II receptors with a dual anti‐ActRIIA/IIB antibody is critical to promote maximal skeletal muscle hypertrophy. Proc Natl Acad Sci U S A 2017;114:12448–12453. 29109273 10.1073/pnas.1707925114 PMC5703284

[35] Marino FE , Risbridger G , Gold E . The therapeutic potential of blocking the activin signalling pathway. Cytokine Growth Factor Rev 2013;24:477–484. 23787160 10.1016/j.cytogfr.2013.04.006

[36] Han HQ , Zhou X , Mitch WE , Goldberg AL . Myostatin/activin pathway antagonism: molecular basis and therapeutic potential. Int J Biochem Cell Biol 2013;45:2333–2347. 23721881 10.1016/j.biocel.2013.05.019

[37] Busquets S , Toledo M , Orpi M , Massa D , Porta M , Capdevila E , et al. Myostatin blockage using actRIIB antagonism in mice bearing the Lewis lung carcinoma results in the improvement of muscle wasting and physical performance. J Cachexia Sarcopenia Muscle 2012;3:37–43. 22450815 10.1007/s13539-011-0049-z PMC3302990

[38] Li Q , Kumar R , Underwood K , O'Connor AE , Loveland KL , Seehra JS , et al. Prevention of cachexia‐like syndrome development and reduction of tumor progression in inhibin‐deficient mice following administration of a chimeric activin receptor type II‐murine Fc protein. Mol Hum Reprod 2007;13:675–683. 17704537 10.1093/molehr/gam055

[39] White JP , Baynes JW , Welle SL , Kostek MC , Matesic LE , Sato S , et al. The Regulation of Skeletal Muscle Protein Turnover during the Progression of Cancer Cachexia in the ApcMin/+ Mouse. PLoS ONE 2011;6:e24650. 21949739 10.1371/journal.pone.0024650 PMC3176277

[40] Johnston AJ , Murphy KT , Jenkinson L , Laine D , Emmrich K , Faou P , et al. Targeting of Fn14 prevents cancer‐induced cachexia and prolongs survival. Cell. 2015;162(6):1365–78. 26359988 10.1016/j.cell.2015.08.031

[41] Wang A , Zhang F , Xu H , Xu M , Cao Y , Wang C , et al. TWEAK/Fn14 promotes pro‐inflammatory cytokine secretion in hepatic stellate cells via NF‐κB/STAT3 pathways. Mol Immunol 2017;87:67–75. 28411440 10.1016/j.molimm.2017.04.003

[42] Hu G , Zeng W , Xia Y . TWEAK/Fn14 signaling in tumors. Tumour Biol 2017;39: 1010428317714624, 101042831771462. 10.1177/1010428317714624 28639899

[43] Hogan KA , Ravindran A , Podolsky MA , Glick AB . The TGFβ1 pathway is required for NFκB dependent gene expression in mouse keratinocytes. Cytokine 2013;64:652–659. 24075100 10.1016/j.cyto.2013.09.004 PMC3942663

[44] Qin H , Chan MW , Liyanarachchi S , Balch C , Potter D , Souriraj IJ , et al. An integrative ChIP‐chip and gene expression profiling to model SMAD regulatory modules. BMC Syst Biol 2009;3:73. 19615063 10.1186/1752-0509-3-73 PMC2724489

[45] Qi Y , Ge J , Ma C , Wu N , Cui X , Liu Z . Activin A regulates activation of mouse neutrophils by Smad3 signalling. Open Biol 2017;7:160342. 28515224 10.1098/rsob.160342 PMC5451541

[46] Fearon KC , Glass DJ , Guttridge DC . Cancer cachexia: mediators, signaling, and metabolic pathways. Cell Metab 2012;16:153–166. 22795476 10.1016/j.cmet.2012.06.011

[47] Scott HR , McMillan DC , Crilly A , McArdle CS , Milroy R . The relationship between weight loss and interleukin 6 in non‐small‐cell lung cancer. Br J Cancer 1996;73:1560–1562. 8664130 10.1038/bjc.1996.294 PMC2074552

[48] Narsale AA , Carson JA . Role of interleukin‐6 in cachexia: therapeutic implications. Curr Opin Support Palliat Care 2014;8:321–327. 25319274 10.1097/SPC.0000000000000091 PMC4323347

[49] Pal M , Febbraio MA , Whitham M . From cytokine to myokine: the emerging role of interleukin‐6 in metabolic regulation. Immunol Cell Biol 2014;92:331–339. 24751614 10.1038/icb.2014.16

[50] Lach‐Trifilieff E , Minetti GC , Sheppard K , Ibebunjo C , Feige JN , Hartmann S , et al. An antibody blocking activin type II receptors induces strong skeletal muscle hypertrophy and protects from atrophy. Mol Cell Biol 2014;34:606–618. 24298022 10.1128/MCB.01307-13 PMC3911487

[51] McPherron AC , Lee SJ . Double muscling in cattle due to mutations in the myostatin gene. Proc Natl Acad Sci U S A 1997;94:12457–12461. 9356471 10.1073/pnas.94.23.12457 PMC24998

[52] Schuelke M , Wagner KR , Stolz LE , Hubner C , Riebel T , Komen W , et al. Myostatin mutation associated with gross muscle hypertrophy in a child. N Engl J Med 2004;350:2682–2688. 15215484 10.1056/NEJMoa040933

[53] Aversa Z , Costelli P , Muscaritoli M . Cancer‐induced muscle wasting: latest findings in prevention and treatment. Ther Adv Medical Oncol 2017;9:369–382. 10.1177/1758834017698643 PMC5424865 28529552

[54] Rooks DS , Laurent D , Praestgaard J , Rasmussen S , Bartlett M , Tanko LB . Effect of bimagrumab on thigh muscle volume and composition in men with casting‐induced atrophy. J Cachexia Sarcopenia Muscle 2017;8:727–734. 28905498 10.1002/jcsm.12205 PMC5659065

[55] Rooks D , Praestgaard J , Hariry S , Laurent D , Petricoul O , Perry RG , et al. Treatment of sarcopenia with bimagrumab: results from a phase II, randomized, controlled, proof‐of‐concept study. J Am Geriatr Soc 2017;65:1988–1995. 28653345 10.1111/jgs.14927

[56] von Haehling S , Morley JE , Coats AJS , Anker SD . Ethical guidelines for publishing in the Journal of Cachexia, Sarcopenia and Muscle: update 2017. J Cachexia Sarcopenia Muscle 2017;8:1081–1083. 29098794 10.1002/jcsm.12261 PMC5700441

