# Supplementary figures and images for: Autocrine activin A signalling in ovarian cancer cells regulates secretion of interleukin 6, autophagy, and cachexia
Source: J Cachexia Sarcopenia Muscle. 2019 Aug 21;11(1):195–207. doi: 10.1002/jcsm.12489 (PMC7015233; doi:10.1002/jcsm.12489)

Figure S1

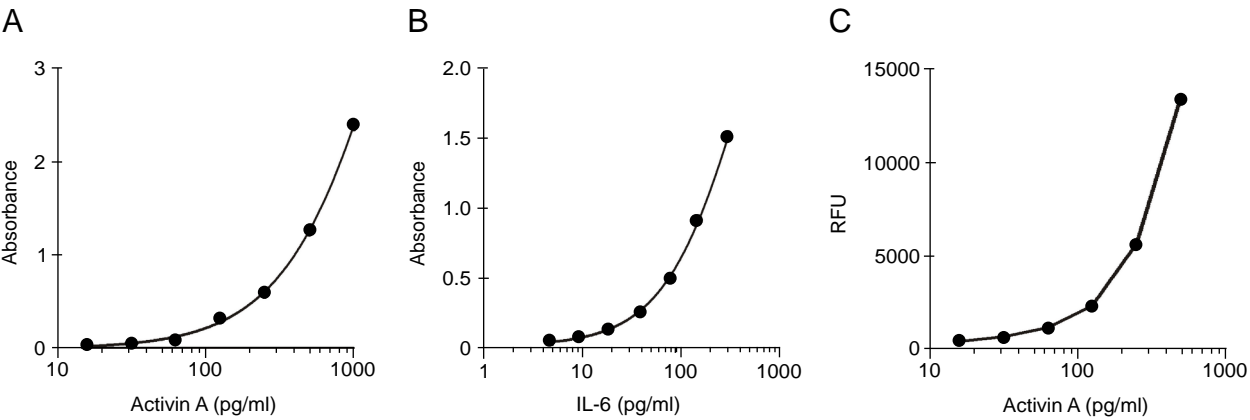

Supplement: Supplementary file 1 — Figure S1. Standard curves from activin A and IL‐6 ELISA. Standard curve for activin A (A) and IL‐6 (B) ELISA used when analyzing TOV21G conditioned medium, and for activin A ELISA used for analyzing serum samples (C). [file JCSM-11-195-s001.pdf]

Figure S2

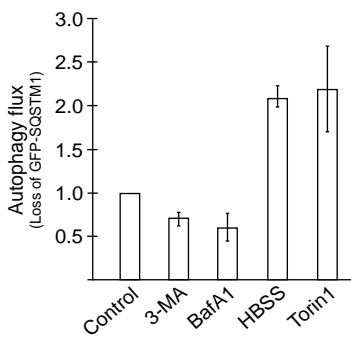

Supplement: Supplementary file 2 — Figure S2. Autophagy flux in autophagy reporter cells treated with autophagy effectors. Autophagy flux in autophagy reporter cells treated for 17 hours with 3‐methyladenine (3‐MA, 3 mM), bafilomycin A1 (BafA1, 100 nM), Hanks' Balanced Salt Solution (HBSS) or Torin 1 (150 nM). [file JCSM-11-195-s002.pdf]

Figure S3

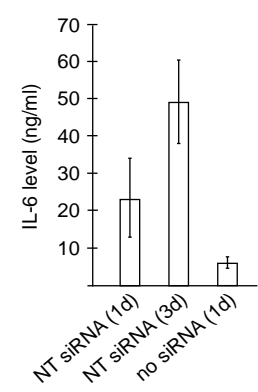

Supplement: Supplementary file 3 — Figure S3. IL‐6 protein level in conditioned medium (CM) from TOV21G cells. Level of IL‐6 in CM from untreated TOV21G cells and TOV21G cells treated with non‐targeting (NT) siRNA. CM from 1 or 3 days post‐seeding (untreated) or post‐transfection (NT siRNA treated). [file JCSM-11-195-s003.pdf]

Figure S4

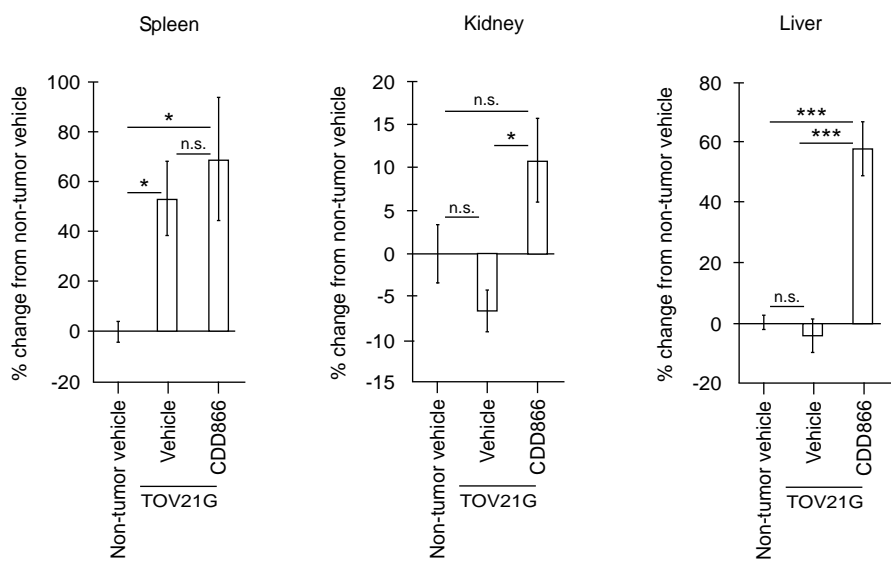

Supplement: Supplementary file 4 — Figure S4. In TOV21G tumor‐bearing mice, injection of the ActRII neutralizing antibody does not affect the weight of the spleen but display an anabolic effect on kidney and liver. Mean relative weight change of spleen, kidney and liver, respectively ±SEM of vehicle‐treated (n = 7) and CDD866‐treated (n = 7) TOV21G tumor‐bearing mice relative to non‐tumor, vehicle‐treated control mice (n = 5). *p < 0.05, ***p < 0.0005 (Student t‐test), n.s. = non‐significant. [file JCSM-11-195-s004.pdf]
